# The Effect of Intraocular Haloperidol on Motor Function in Models of Two Neuropsychiatric Disorders: Implications for the Origin and Treatment of Parkinson’s Disease, Psychosis and Drug Addiction

**DOI:** 10.3390/brainsci15101062

**Published:** 2025-09-29

**Authors:** Gregory L. Willis

**Affiliations:** The Bronowski Institute of Behavioural Neuroscience, Woodend, VIC 3442, Australia; director@bronowski.org

**Keywords:** Parkinson’s disease, haloperidol, L-dopa, retina, amphetamine, circadian

## Abstract

**Background:** It has recently been proposed that the retina plays an important modulatory role in the control of motor function that is usually attributed exclusively to the function of the nigro-striatal dopamine (NSD) system. Indeed, it has been proposed further that Parkinson’s disease (PD) begins in and progresses from the retina and may be effectively treated from there. While previous intraocular work has employed intravitreal (IVIT) administration of toxins to induce experimental PD, the first study series reported here examines the effect of IVIT haloperidol on motor performance while the second study examines the effect of IVIT haloperidol on the unilateral rotation model of PD, both in a circadian context. **Methods:** Motor tests included open field performance and the latency to perform three motor tests after the IVIT injection of haloperidol with and without amphetamine pretreatment. In a second study, IVIT injections of the melatonin antagonist ML-23 or L-dopa were made after unilateral lesions of the NSD in rats that were placed in a rotometer examining spontaneous ipsilateral and contralateral turning. **Results:** IVIT haloperidol produced robust changes in several motor parameters during the light and dark phase of the LD cycle which were enhanced by amphetamine pretreatment. In the second study, while IVIT L-dopa had only a minor effect on spontaneous rotation during the light phase, IVIT haloperidol produced a robust effect upon ipsilateral turning. The reduction in spontaneous ipsilateral turning was seen after IVIT injections into the eye ipsilateral or contralateral to the hemisphere in which NSD destruction occurred. Reduced turning was seen during both the light and dark phases of the L/D cycle. **Conclusions:** These results illustrate that IVIT injections of DA and melatonin receptor antagonists can differentially alter motor function via the retina. This suggests that the retina may be a treatment target not only for PD but also for other DA- and melatonin-mediated disorders such as drug addiction, psychosis and schizophrenia.

## 1. Introduction

It has recently been suggested that the retina modulates regulatory and motoric function in Parkinson’s disease (PD) via its link to anatomically specific deep brain systems [1]. This hypothesis has been reconfirmed in preclinical and clinical studies implementing intravitreal (IVIT) injections of anti-Parkinsonian drugs in animal models bearing dopamine (DA)-depleting lesions and in clinical studies where light treatment has been implemented as a treatment to restore circadian function [2]. These findings, taken in the context of concomitant nigro-striatal and retinal DA loss [3,4,5,6], suggest that other approaches to DA replacement may be effective in PD. Indeed, approaches whereby visual function with non-invasive [7,8,9] or surgical intervention [10,11] intimate a functional connection between visual and motor systems are worthy of further exploration. In fact, recent speculation that the retina provides a more viable route of access for treating neurological disease suggests that PD may well be a disorder in which the retina plays a prominent role [12]. In previous work, this approach has been presented in a circadian context whereby DA and melatonin sit in functional opposition, with DA predominating during the day and melatonin being functionally active at night. In PD this intricate balance is compromised with the NSD system and the retina becoming depleted of DA, rendering melatonin levels disproportionately elevated. To examine the retinal/NSD relationship further [1] minute doses of IVIT L-dopa, or the melatonin antagonist ML-23, were administered to Parkinsonian rats during the light or dark phase of the light/dark (L/D) cycle. The doses employed were so minute the possibility of leakage to the cerebral compartment was remote. As hypothesized, IVIT L-dopa improved motor and vegetative function during the light phase while ML-23 did this mainly during the dark phase of the L/D cycle. This supported the notion that the circadian system may play an important role in PD and that the intravitreal approach to treatment may be an effective means of targeting the retina and treating PD.

Further support for this hypothesis is derived from studies demonstrating the ocular effects of DA replacement after systemic administration. For example, L-dopa has been shown to repair visual function after systemic injection [13] while simultaneously repairing motor control. Such findings are consistent with other studies where systemic, dopamine antagonist administration with haloperidol [14] concomitantly produce changes in retinal and motor function typical of those seen in PD [15,16,17,18,19,20]. These findings further support the contention that the retina plays a role in the etiology and treatment of PD.

To further explore aspects of this hypothesis, we administered a DA receptor antagonist via the IVIT route to determine if it could render motoric and psychotropic effects in a routine model of PD. Better understanding of this concept, particularly in light of recent findings [1,2,21], might prove beneficial in two regards: First, given that drug-induced PD can occur after systemic haloperidol [14,22], it would be useful to determine if the site of action might include the retina. Secondly, if the retina plays a role in drug-induced effects then the retina, as a therapeutic target, should be examined. To explore these two questions, we have undertaken two experimental approaches. The first examined the role of DA receptor antagonists in their capacity to produce PD [22,23,24] and to treat psychosis after DA replacement [24,25]. The second investigates the role of IVIT drugs in the treatment of PD using the rotational model of PD in rodents commonly employed for preclinical testing of potential pharmacotherapies [26,27,28]. To examine the possible contribution of circadian function, all dependent variables were examined during the light and dark phases of the light/dark (L/D) cycle in both studies.

## 2. Materials and Methods

### 2.1. Animals

For Study 1, 27 male Sprague Dawley rats were obtained from the Bronowski Institute colony and were housed individually in wire mesh cages with standard food pellets (Clarke King^®^/Barastock^®^, Kyneton, VIC, Australia) made available ad lib from a feeding grid positioned on the roof of the cage. Tap water was made available from bottles attached to the top of each cage. Animals ranged in weight from 250 to 350 Gms at the commencement of the experiment. Room temperature was maintained at 22 °C ± 2 °C with a 12 h light/dark (L/D) cycle with lights on at 07:00 h. The room was illuminated with 4 fluorescent tubes with the intensity of light within each cage averaging 250 lux during the light phase of the L/D cycle. No light was detected in the housing facility during the dark phase.

In Study 2, 14 experimentally naive male Sprague Dawley rats were obtained, housed, fed and maintained in the same manner as that described for Study 1. As described in Study 1, all experiments were performed under the auspices of the Animal Experimentation Ethics Committee of the Bronowski Institute of Behavioural Neuroscience.

### 2.2. Study 1

#### 2.2.1. Design of Study 1

After habituation into the colony for at least 7 days, all animals were handled by all experimenters prior to commencing the formal part of each study. In the first experiment in Study 1, there were two groups consisting of 7 animals per group, with the first group receiving a bilateral IVIT haloperidol injection while the second group received an IVIT injection of vehicle. In the second experiment, there were 7 animals in the IVIT-haloperidol-injected group and 6 in the vehicle-treated group, similar to the design of study one. However, in the second experiment, for 5 days prior to the first IVIT injection, all rats were injected with 1 mg/kg of dl amphetamine twice daily at 10:00 and 17:00 h. In both experiments, rats were tested 3 h after the IVIT injection during the light phase of the L/D cycle, with testing commencing at about 10:00 h. Approximately 36 h later all animals were again tested just after the onset of the dark phase (at ≈ 20:00 h), 3 h after receiving a second IVIT injection of their respective drug. Further details of the IVIT injections are described subsequently and the 5 tests of motor function are described later in this section.

#### 2.2.2. Drugs and Solutions for Intraperitoneal (I.P.) and IVIT Injections

Haloperidol was acquired from Sigma-Aldrich (St. Louis, MO, USA), and was prepared for IVIT injection at a concentration of 1 μg/μL by dissolving it in a sterile 70% DMSO solution while vehicle injections were made with 2 µL of this carrier solution. Dl-amphetamine was acquired from Sigma Chemicals ([+/−]-α-methyl-amphetamine; St. Louis, MO, USA) and mixed in a concentration of 0.5 mg/mL for the delivery of 1 mg/kg of bodyweight. New solutions of the drug were prepared immediately prior to injection with stock solutions kept refrigerated or on ice and shielded from light. All solutions were discarded immediately at the end of each injection session. All concentrations for IVIT administration of the drug were chosen on the basis of previous work describing a localized effect of similar compounds when injected by the intracerebral route [1,29,30]. These reports suggest that the observed effect does not merely represent leakage of test substances into sensitive brain areas after IVIT administration.

#### 2.2.3. I.P. and IVIT Injections

I.P solutions of amphetamine were carefully administered without stressing the animal by gently holding them in a supine position. Experimenters were experienced and no signs of stress were evident during the injection procedure. All animals were placed back into the holding cage immediately after injection awaiting testing.

Injections into the vitreous humour [1] were made with the aid of a 10 μL syringe fitted with a 26 g needle that was 75 mm in length. The needle was fitted with a coloured plastic sleeve exposing 3 mm of the tip to allow the experimenter to gauge the depth of needle for insertion into the centre of the vitreal mass. Rats were first placed in a clear Perspex induction chamber 200 × 300 × 400 mm fitted with a base constructed from heavy-gauge plastic netting overlying a 125 mm layer of cotton fibre that served to absorb and hold the anesthetic. Isoflurane inhalation anesthetic (Attane-Bomac: 1 mL/mL) was employed by placing approximately 10–20 mL into the absorbable cotton surface just prior to placing the animal into the induction chamber. Exposure of the animal for 60 to 100 s induced a state of deep anesthesia that lasted 40 to 60 s, thereby permitting the injection of the test substances and vehicle bilaterally into the vitreous. To keep the preparation clean, the fur was swabbed with 70% ETOH for cleansing the surrounding area prior to injection. To facilitate injection into the lateral aspect of the eye, light pressure was placed on the caudal surface of the eye using a sterile, gloved, tip of the index finger to cause it to become a maneuverable exophthalmic mass and thereby permitting the gentle application of counter-pressure when the needle was inserted into the vitreous mass. Haloperidol was injected bilaterally in a volume of 2 μL, commencing with the left eye receiving the first injection, as a matter of orderly procedure. Vehicle injections were made in the volume of 2 μL. When injections were complete, the area was gently swabbed with sterile, isotonic saline and a drop of antibiotic ointment (Amacin^®^ eye and ear ointment), and was placed on the cornea as a prophylactic. Rats were held and kept warm until they were able to ambulate on their own and then each returned to their individual cage, which took an average of 60 s. A period of ten days was permitted between consecutive IVIT injections when testing during different phases of the light/dark (L/D) cycle. This method has been utilized and described previously [1].

#### 2.2.4. Behavioural Measures

Independent variables were measured during the light and the dark phase of the L/D cycle, commencing between the hours of 10:00 and 15:00 h and again at 20:00–01:00 h, respectively, with at least 18 h allowed between consecutive measurements.

Locomotion and rearing were measured with the aid of a 900 mm (length) × 500 mm (width) × 300 mm (Height) PVC box fitted with machine vision with motion detection capabilities, with this system used extensively for quantifying open field behaviour [1,31]. The total number of movements within the horizontal plane and the number of rearing-associated movements in the vertical plane during each 10 min test session were measured and recorded with the aid of specialized software. Due to the high level of variability between subjects, the parameters of horizontal and vertical movement were calculated by subtracting the test scores from the pre-test control scores for each animal, and these were used for analysis. A series of three motor reflex tests were performed immediately at the conclusion of the open field test [32]. These tests included the latency to retract the left, then the right, front limbs when they were elevated 25 mm from the table surface, the latency to step up or down from a raised platform when the rear torso was elevated 25 mm and the latency to ambulate outside of a 90 × 170 mm rectangle drawn on the bench surface. These tests are derivations of those described previously and have been used extensively to characterize the features of experimental PD. The test chamber and all surfaces and each apparatus were thoroughly washed between the testing of each animal to avoid contamination which may cause distraction during testing. Testing during the dark phase of the L/D cycle was performed under low-intensity red light, with all sources of illumination masked by implementing red barrier filters.

### 2.3. Study 2

#### 2.3.1. Design of Study 2

After surgery at least 10 days were allowed before commencing the formal part of the experiment. During this time all animals were handled by the experimenters to ensure that they were habituated to human contact. In the first study, all animals were assigned to either one of two groups, drug or vehicle. They were then injected with 6-OHDA through the in-dwelling cannulae, as described immediately below. After 10 days all animals were placed in the rotometer and their tendency to spontaneously rotate ipsilaterally or contralaterally during a 10 min test period was recorded, and this served as the baseline measurement. These control measurements were first taken immediately after the onset of the light phase of the light/dark cycle (commencing ≈ 09:00 h), or just after the onset of the dark phase (commencing ≈ 20:00 h). At least 5 weeks later, 7 animals received an ipsilateral Intravitreal (IVIT) injection of ML-23 while the 6 remaining animals in the control group received an IVIT injection of vehicle ipsilateral to the intracranial injection during the light phase of the L/D cycle. All animals were tested in the rotometer 3 h after the IVIT injection. After allowing at least 3 days between injections, this procedure was repeated with the exception that in a cross-over design those injected with ML-23 were now injected with vehicle and vice versa. This procedure was then repeated for the dark cycle, allowing at least 3 days between IVIT injection and testing. The entire paradigm was replicated using L-dopa as the injectate for both the light and dark phases. During testing, rats were again placed in the rotometer and their tendency to rotate spontaneously in an ipsilateral or contralateral direction during a 10 min test period was recorded. After raw scores were collated, the ratio of ipsilateral to contralateral rotations made during the 10 min test session were calculated.

#### 2.3.2. Surgery

After habituation into the colony for at least 7 days, rats were pre-medicated with atropine sulphate (0.06 mg/kg-S.C.) and then anesthetized with a Ketamine^®^ (55 mg/kg)/Xylazine^®^ (20 mg/kg) mixture (I.M.). When full anesthesia was achieved, each rat was then placed in a stereotaxic instrument. The site of cannulation for eventual intracranial (I.C.) injection for achieving experimental PD was the posterior lateral hypothalamus (PLH) [32] just rostral to the midbrain diencephalon border in the bundle of NSD system fibres. A 23-gauge stainless steel cannula was implanted on one side of the brain just dorsal to the intended site of injection at the coordinates AP = −1.8 mm; L = ±1.8 mm; D = −6.1 mm. Half of the animals in each group were implanted unilaterally on the left side while the other half here implanted on the right. This site was chosen so that the injection needle extended 2 mm beyond the cannulae tip in a ventral direction to minimize damage to the injection site. All coordinates were relative to bregma and in the plane of Pellegrino et al., 1979 [33]. This position has been found to be effective in producing severe Parkinsonian-like effects in rats [31,32]. At the completion of intracranial surgery, rats were injected with 12 mL/kg Reversine^®^ (S.C.), which was used as a reversal agent for the Xylazine^®^. All rats were injected with the analgesic Meloxacam^®^ (10 mg/kg, I.M.) at the completion of surgery. Rats were kept warm after surgery and then allowed at least 10 days of recovery before commencing the formal part of this study.

#### 2.3.3. Drugs and Solutions for Intracerebral and IVIT Injections

Intracerebral injections [1] were made with 6-hydroxydopamine hydrobromide (Sigma, St. Louis, MO, USA). 6-OHDA was mixed in a concentration of 8 μg/μL and injected in a volume of 2 μL per site. It was dissolved in saline ascorbic solution to prevent rapid oxidation of the drug [32]. New solutions of the drug were prepared immediately prior to injection, with stock solutions kept refrigerated or on ice until used. All solutions were kept shielded from light and then discarded immediately at the end of each injection session.

L-dopa (HCl) for IVIT injection was obtained from a commercial source (Sigma-Aldrich, St. Louis, MO, USA) and was dissolved in isotonic saline to achieve a 100 mM solution. Control injections for the L-dopa study were made with isotonic saline. ML-23 was synthesized by AMRAD Pharmaceuticals and dissolved in a 70% DSMO solution to achieve a 10 mM concentration. Control injections for this study were made with a 70% DSMO solution. Due to the insolubility of ML-23, DSMO was necessary to get ML-23 into solution, and the potential toxic effects were taken into consideration, with the doses of drug employed for IVIT injection selected on the basis of previous work [1,32] and in consideration of previous work describing a localized effect of similar compounds when injected by the intracerebral route [1,29,30]. This suggests that any observed effects are unlikely to represent leakage of test substances into brain areas after IVIT administration.

#### 2.3.4. Intracerebral and IVIT Injections of ML-23 and L-Dopa

Intracerebral Injections with 6-OHDA or isotonic saline were made at a rate of 1 μL per min and the needle was left in situ for at least 30 s after each injection was complete to ensure that the drug diffused from the end of the needle. Each animal was then tested at intervals as described in Study 1.

Injections into the vitreous humour with L-Dopa and ML-23 were also performed as described in Study 1.

#### 2.3.5. Behavioural Measures

During testing in the rotometer, each rat was placed in a bole-shaped device for a 10 min test period. The harness was constructed from of a piece of adjustable Velcro and was fitted just below the rib cage, and the electronic recording device counted the number of clockwise and counter-clockwise rotations in a manner similar to that described previously [21]. These were later translated to ipsi- or contralateral rotations in relation to the side where intracerebral and IVIT injections were made. Spontaneous turning was measured during the light phase and the dark phase of the light/dark cycle, and no systemic drugs were administered during these sessions to induce turning. Due to the subtlety of the IVIT injection there was concern that large systemic doses of DA drugs typically used to induce rotation would mask any effects produced by the IVIT injections. The number of turns was determined in relation to the side of the brain receiving intracerebral injection, and this was translated to ipsi- and contralaterality.

All surfaces within the rotometer were thoroughly washed between the testing of each animal to avoid contamination which may cause distraction during testing.

Body weight was measured periodically after testing in the rotometer between 4 and 8 weeks after intracranial injection. Testing during the dark phase of the light/dark cycle was performed under low-intensity red light or by applying red barrier filters to all sources of illumination.

### 2.4. Statistical Analysis

For Study 1, statistical analysis was undertaken using the SPSS 11.0 for Windows. Due to the skewed distribution and non-homogenous variance which typically occurs with the implementation of these behavioural measures and how the low number of animals per group violates basic assumptions of parametric evaluation, non-parametric testing was chosen to evaluate differences between independent groups and to minimize type II errors. On this basis, the Mann–Whitney-U Test (MWUT) was performed between haloperidol- and vehicle-treated groups for Study 1 and for the haloperidol plus amphetamine- versus vehicle-treated groups for Study 2. Given that the hypothesis permitted prediction of the direction of the expected outcome, a one-sided test was employed with exact significance. The confidence levels were chosen a priori and set at 0.05 to depict a significant effect while confidence levels ranging from 0.051 to 0.099 depicted a trend.

For Study 2 the same statistical package was used to permit comparisons between ipsilateral and contralateral turns for the ML-23 and L-Dopa drug-treated and vehicle-treated groups, performed using one way ANOVA with a Time by Group analysis. The confidence levels were chosen a priori and set at 5% to depict a significant effect while confidence levels ranging from 0.051 to 0.099 were regarded as a trend. Due the exploratory nature of this study and the small number of observations employed, a conservative interpretation of any trends that occurred were noted.

## 3. Results

### 3.1. Study 1

**Figure 1:**
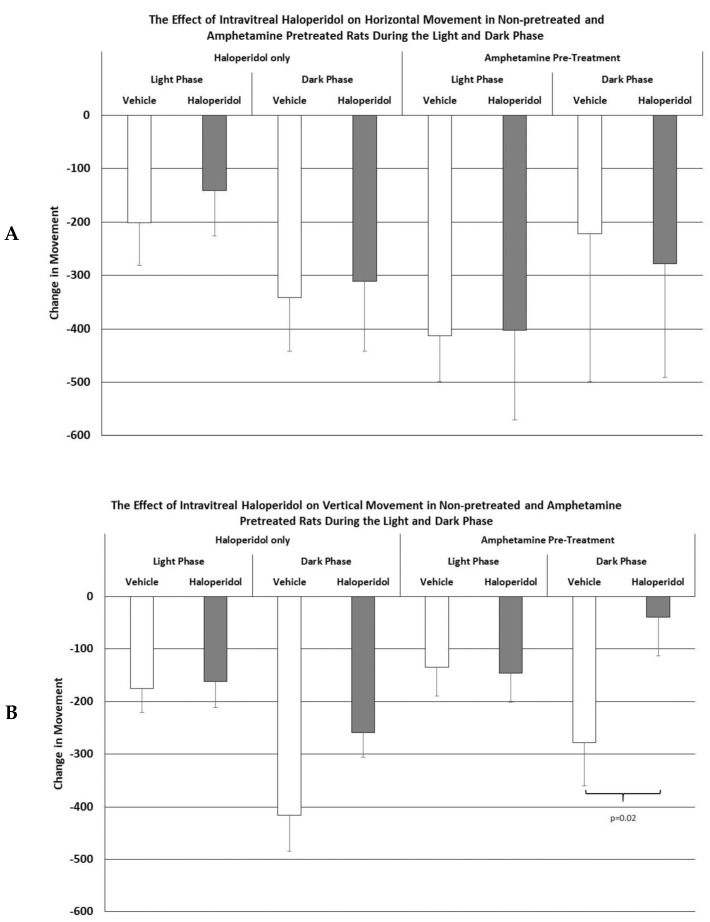
The mean change in horizontal (**A**) and vertical (**B**) movement in normal and amphetamine-pretreated rats after IVIT injection 3 h prior to testing. Rats were tested just after the onset of either the light or dark phase of the L/D cycle. The inclusion bar shows the groups that were compared for statistical comparison and the *p* value shows the level of significance obtained for each comparison. Significant effects were derived from *p* values of *p* ≤ 0.05, while significant trends were defined as *p* values ranging from 0.051 to 0.099. The T-bars represent the standard error of the mean.

As shown in Figure 1, the IVIT injection of haloperidol into normal or amphetamine-pretreated rats had no effect upon horizontal movement during the light or dark phase of the L/D cycle. Similarly, while there were no significant differences in vertical movement in normal rats, vertical movement during the dark phase was significantly affected after the intravitreal injection of haloperidol, which was about a 6 fold improvement over controls (*p* < 0.02).

**Figure 2:**
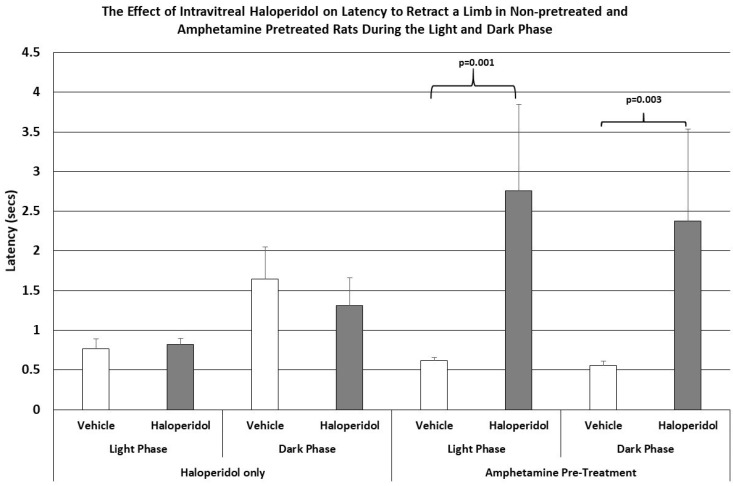
The mean latency to retract an elevated limb in normal and amphetamine-pretreated rats after IVIT injection 3 h prior to testing. Rats were tested just after the onset of either the light or dark phase of the L/D cycle. The inclusion bar shows the groups that were compared for statistical comparison and the *p* value shows the level of significance obtained for each comparison. Significant effects were derived from *p* values of *p* ≤ 0.05 while significant trends were defined as *p* values ranging from 0.051 to 0.099. The T-bars represent the standard error of the mean.

Similarly, as shown in Figure 2, the latencies to retract an elevated limb during the light or dark phase of the L/D cycle in rats receiving IVIT haloperidol had no effect upon this parameter. However, when haloperidol-injected rats were pretreated with amphetamine the, ability to retract a limb was significantly impaired, increasing the latency to perform this task by a magnitude of 3 to 4 times that of control levels during the light phase (*p* = 0.001) and the dark phase (*p* = 0.003) of the L/D cycle.

**Figure 3:**
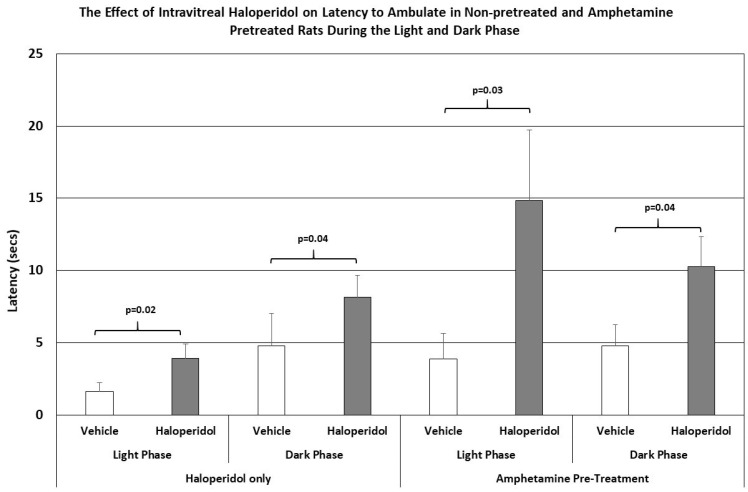
The latency to ambulate from a prescribed area in normal and amphetamine-pretreated rats after IVIT injection 3 h prior to testing. Rats were tested just after the onset of either the light or dark phase of the L/D cycle. The inclusion bar shows the groups that were compared for statistical comparison and the *p* value shows the level of significance obtained for each comparison. Significant effects were derived from *p* values of *p* ≤ 0.05, while significant trends were defined as *p* values ranging from 0.051 to 0.099. The T-bars represent the standard error of the mean.

The latency to step up or down was not affected by IVIT injection of haloperidol in either group. However, the latency to ambulate was more severely affected in all groups and at all test times (Figure 3). The latency to ambulate from within a prescribed area was significantly slowed in haloperidol-injected animals compared to those injected with vehicle. This was the case for testing during both the light phase and the dark phase of the L/D cycle (*p* = 0.02 and *p* < 0.04, respectively). The latency to ambulate was also severely impaired in the amphetamine-pretreated group, with the observed differences occurring during the light and dark phases being highly significant (*p* < 0.03 and *p* = 0.04, respectively). The magnitudes of change in latency were again greater in the amphetamine-treated group than in normal rats.

### 3.2. Study 2

**Figure 4:**
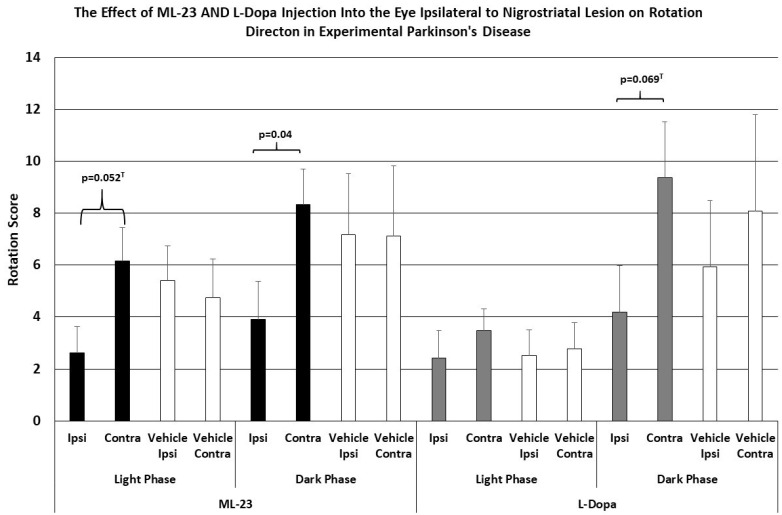
The mean change in spontaneous rotation in rats after IVIT injection of L-dopa, ML-23 or vehicle into the ipsilateral eye 3 h prior to testing. Rats were tested just after the onset of either the light or dark phase of the L/D cycle. The inclusion bar shows the groups between which statistical comparisons were made and the *p* value shows the level of significance obtained for each comparison. Significant effects were derived from *p* values of *p* ≤ 0.05 while significant trends were defined as *p* values ranging from 0.051 to 0.099. The T-bars represent the standard error of the mean.

As shown in Figure 4, the intraocular injection of ML-23 in microgram quantities alters spontaneous rotation caused by the unilateral administration of the neurotoxin 6-OHDA. In injection of ML-23 into the vitreous ipsilateral to the lesion (left trace) there was a trend toward reduced ipsilateral turning with less than half of the contralateral turns during the light phase (ANOVA, df = 1, 13; F = 4.666, *p* = 0.052). Similarly, during the dark phase of the L/D cycle, spontaneous ipsilateral turning was significantly reduced (ANOVA; df = 1, 3; F = 4.889, *p* = 0.047). Note that contralateral and ipsilateral turning were not significantly different in the IVIT controls during the light (ANOVA; df = 1, 13, F = 0.110, *p* = 0.746) or the dark phase (ANOVA; df = 1, 13, F = 0.000, *p* = 0.988).

As also shown in Figure 4 (right trace), no significant change in spontaneous ipsi- vs. contralateral rotation was seen after IVIT injection of L-dopa ipsilateral to the NSD lesion during the light phase (ANOVA; df = 1, 13, F = 0.035, *p* = 0.854), and this was similar to the lack of effect seen after the injection of vehicle (ANOVA; df = 1, 13; F = 0.000, *p* = 0.988). There was a trend indicating a tendency to increase contralateral turning, compared to ipsilateral rotations, after L-dopa was administered during the dark phase (ANOVA; df = 1,13, F = 0.983, *p* = 0.069), but no difference was seen in vehicle-injected controls during the light or dark phase (ANOVA; df = 1, 13, F = 0.287, *p* = 0.615).

**Figure 5:**
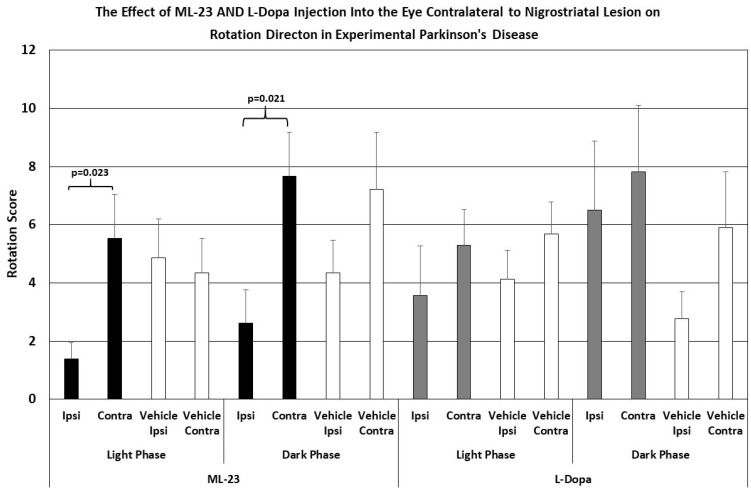
The mean change in spontaneous rotation in rats after IVIT injection of L-dopa, ML-23 or vehicle into the contralateral eye 3 h prior to testing. Rats were tested just after the onset of either the light or dark phase of the L/D cycle. The inclusion bar shows the groups between which statistical comparisons were made and the *p* value shows the level of significance obtained for each comparison. Significant effects were derived from *p* values of *p* ≤ 0.05 while significant trends were defined as *p* values ranging from 0.051 to 0.099. The T-bars represent the standard error of the mean.

As shown in Figure 5, the intraocular injection of ML-23 also altered spontaneous turning after the unilateral administration of the neurotoxin 6-OHDA into the NSD system. Injection of ML-23 into the eye contralateral to the lesion (left trace) caused a reduction in ipsilateral turning which was 1/3 the number of contralateral turns caused by the IVIT injection during the light phase (ANOVA, df = 1, 13; F = 6.761, *p* = 0.023). Similarly, the injection of ML-23 into the eye contralateral to the NSD lesion again caused a significant decrease in ipsi- versus contralateral turns that was also significant during the dark phase (ANOVA; df = 1, 3; F = 7.096, *p* = 0.021). Contralateral and ipsilateral turning was not significantly different in the IVIT controls during the light (ANOVA; df = 1, 13, F = 0.077, *p* = 0.786) or the dark phase (ANOVA; df = 1, 13, F = 1.617, *p* = 0.228).

Figure 5’s right trace shows no significant difference in ipsi- versus contralateral rotation after IVIT injection of L-dopa contralateral to the NSD lesion when performed during the light cycle (ANOVA; df = 1, 13, F = 0.793, *p* = 0.391). This lack of effect was similar to that seen after the injection of vehicle (ANOVA; df = 1, 13; F = 1.286, *p* = 0.279). Neither was there any difference in ipsilateral turning versus contralateral turning after L-dopa was administered in the eye contralateral to the NSD lesion during the dark phase (ANOVA; df = 1, 13, F = 0.187, *p* = 0.673). Vehicle-injected controls also failed to show any difference in ipsi- versus contralateral turning after injection into the contralateral eye during the dark phase (ANOVA; df = 1, 13, F = 2.150, *p* = 0.139). In other words, ipsilateral turning after IVIT injection of L-dopa on the side ipsilateral to NSD destruction was not observed (cf. Figure 1, right trace)

**Figure 6:**
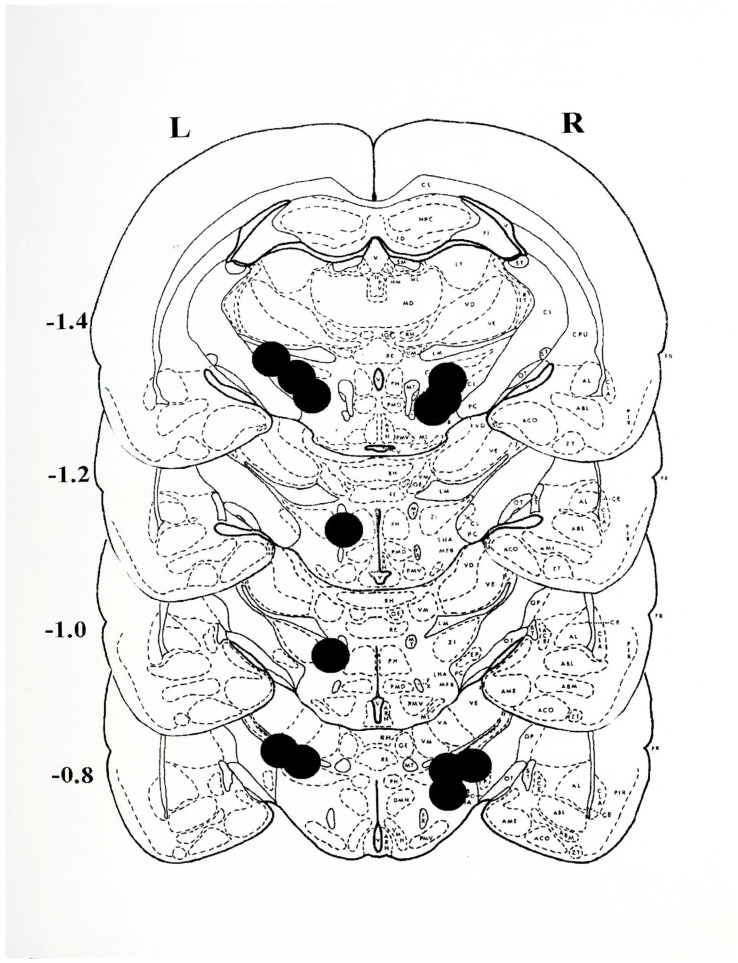
Histological plates from Pellegrino et al., 1979 [33], depicting the sites of 6-OHDA injection. The approximate centre point of each injection was located on plates extending from −0.8 to −1.4 mm posterior to bregma for animals receiving a single, unilateral injection on the left (L) or right (R) side of the brain. The lesions were found to extend approximately 1 mm anterior and posterior to the depicted sites. Anatomically, the lesions extended dorsally from the medial lemniscus (LM) to the most ventral position into the lateral hypothalamus (LH) and into the adjacent medial forebrain bundle (MFB). The most lateral extension of the lesions encroached upon the medial margins of the internal capsule (CI) and medially to the external margins of the mammillothalamic tract (MT). Note that the sites of injection depict the nonspecific damage resulting from the injection of the neurotoxin 6-OHDA, and that in all cases the positioning permits the diffusion of the drug so as to permit assess to the fibres of passage of the nigro-striatal dopamine system.

The histological plates shown in Figure 6 illustrate the location of nonspecific damage caused by the unilateral intracerebral injection of 6-OHDA to induce unilateral experimental PD.

## 4. Discussion

### 4.1. IVIT Haloperidol and L-Dopa and Amphetamine Pretreatment

Results from Study 1 demonstrate that the IVIT administration of minute doses of haloperidol significantly slow motor performance with and without systemic amphetamine pretreatment. Recent studies have shown that minute quantities of neurotoxins administered via the IVIT route can induce experimental PD, thereby supporting the concept that the eye plays an important role in the etiology of PD and that the retina, as part of the circadian system, may be intimately involved [12]. This has been demonstrated in a previous study that delivered microgram quantities of anti-PD drug via the IVIT route to rats bearing lesions of the NSD system and this repaired experimental PD [1]. From this we deduced that other classes of drug affecting the DA system would have similar effects when administered by the IVIT route. For this reason haloperidol was selected on the basis of its action as a DA receptor antagonist commonly employed as an antipsychotic that can produce drug-induced PD. This makes haloperidol an ideal candidate for exploring the retina/PD hypothesis [22,23,24]. Results presented in the first study demonstrate that haloperidol administered by the IVIT route impairs motor performance. Furthermore, this is also consistent with the clinical reports that haloperidol not only causes extrapyramidal symptoms and enhances PD [14,15], but that the combination of IVIT haloperidol plus amphetamine enhances the Parkinsonian symptoms reported to occur with amphetamines alone [34]. This suggests further that the retina may be an important etiological factor for drug-induced PD, for the idiopathic form of PD [3,35,36] and in drug addiction. The most surprising find was that the IVIT doses employed are only a fraction of those required to evoke a similar response with systemic administration. Clinical implementation of this would dramatically reduce or even avoid adverse effects resulting from systemic DA replacement or for treating drug addiction [1].

Note that all motor parameters examined do not respond equally to IVIT drugs or toxins as reported in other studies [32]. Although horizontal and vertical movement were only minimally affected by IVIT haloperidol, there were robust effects observed on motor reflex control, including latency to retract a limb and to ambulate. This may be a dose-dependent effect whereby indices of finer motor control are affected by lower dosing achieved by IVIT administration. Even though the IVIT doses chosen here were selected on the basis of previous studies employing intracranial injections [29], further exploratory studies using larger IVIT doses are currently underway. It is also important to note that the observed reductions in activity seen in amphetamine-pretreated rats are induced by a fraction of the much larger doses routinely administered systemically to achieve the same effect [14,37,38]. This is consistent with studies demonstrating that the blockade of DA receptors with low-dose haloperidol enhances DA receptor sensitivity and that this process may mediate the increased response of PD patients to DA replacement [34]. The improvement in vertical movement was 6 fold over that of controls, and this confirms that low doses of haloperidol administered to the retina exert a potent effect. This is consistent with the very early hypothesis that DA receptor antagonists administered prior to DA-depleting lesions induce DA receptor hypersensitivity that can facilitate recovery from experimental PD [39]. This is also consistent with the behavioural recovery seen with the peripheral DA receptor antagonist domperidone that facilitates recovery from experimental PD [40] but does so via peripheral DA receptor blockades [41,42]. We hypothesize that retinal DA receptors may participate in the recovery from DA deficiency-mediated deficits. The function of the retina, its subparts and its interaction with various brain structures is the subject of ongoing exploration with the purpose of addressing these interesting possibilities.

### 4.2. IVIT Haloperidol and the Rotation Model

Results from the PD rotation model study show that minute IVIT doses of L-Dopa and the melatonin antagonist ML-23 that can significantly alter rotational behaviour via an effect mediated by the retina.

Results from the IVIT/rotation study provide confirmation of the hypothesis implicating an active role for the retina in motor control. That minute quantities of anti-Parkinsonian drugs can alter the turning response after IVIT delivery corroborates previous pilot work showing that ipsilateral or contralateral enucleation significantly alters the turning response in rats with unilateral lesions of the NSD system when they are accompanied by enucleation [21]. This is an important finding in relation to the use of the rotation model as a routine, preclinical tool for screening potential candidates for novel anti-Parkinsonian drugs. It illustrates the importance of the visual system in the expression of PD and highlights the involvement of anatomical connections between central DA systems and the retina that may be affected in PD [1]. In this regard, a critical re-evaluation of the core preclinical drug testing in this context would help to better define the value of unilateral rotation model as a screening tool.

It is interesting to note the difference in the ratio of ipsilateral to contralateral turning in vehicle-injected rats during the light phase versus the dark phase of the light/dark cycle. When IVIT injections were made with ML-23, contralateral turning was most robust, regardless of the L/D phase, or which eye was injected. The turning response after IVIT L-dopa was weak and was only seen during the dark phase when the eye ipsilateral to the NSD lesions was injected. Diurnal differences in motor performance when melatonin versus DA function are challenged at different times of the L/D cycle have been observed in previous studies [1]. This is consistent with the proposition that these two systems function interdependently to control motor performance [12] and that the cells of the retina, the pineal and the substantia nigra are functionally dependent upon a balance between melatonin and dopamine. This reciprocation is functionally tied to the day night cycle, which may be clue as to how these systems might be functionally compromised in PD and other neurological disorders.

This tendency to show a turning response that favours ipsilateral tuning after IVIT melatonin in the unilateral 6-OHDA model was an unexpected finding. In previous work, ipsilateral turning was increased in the presence of enucleation on the same side as a unilateral lesion of the NSD [21]. Conversely, IVIT injection of the melatonin antagonist ML-23 decreased ipsilateral turning after injection into either the ipsilateral or contralateral eye. This illustrates how the input from a retinal-based system can influence motoric function compared to the minimal effect observed with IVIT L-dopa. From a scientific perspective, it is difficult to fully understand the mechanism(s) by which the primary visual, accessory optic and retino-hypothalamic systems participate in the observed phenomena. Our basic scientific and clinical work is examining the efficacy of several methodological approaches for altering circadian and autonomic function to help clarify more effective treatment strategies to compliment DA replacement.

The models implemented in the present study have limitations in regard to the generalizability to their clinical application in PD. For example, in the bilateral model the acute onset of symptoms does not approximate the insidious features of the clinical syndrome. In fact, after induction of PD in both the bilateral and rotational models there is a course of spontaneous recovery which is the antithesis of the progressive deterioration experienced by PD patients. Similarly, the use of the turning model inserts post-induction sequelae of pharmacologically inducing artefacts that only remotely reflect the neurochemical challenges characterizing the disease itself. For this reason a conservative interpretation of the present findings is recommended until further work progresses.

Other limitations of the present study include methodological issues such as the use of dilute DMSO as a vehicle for IVIT injection [43] and its toxicity when used in local injections, which may have complicated the interpretation of results. Future studies employing other vehicles as well as systemic injections to serve as controls for the ‘ocular only’ effect of the IVIT injections should be undertaken. However, it is important to keep in mind that the doses of L-dopa, ML-23 and haloperidol used in the present study are several magnitudes lower than those implemented in previous work [1,14,30] to evoke a lesser change in the dependent variables.

It has become clear that the simple replacement of deficient DA does not address all features that manifest in these models. Novel findings such as those presented here can provide new directions for a disease that is locked in a paradigm with many limitations. Substantial evidence is now emerging demonstrating that the visual system may play a major role in the etiology and progression of PD. This novel approach may provide the opportunity to substantially improve the quality of life of patients where DA replacement has failed.

## 5. Conclusions

The present results build on previous findings, suggesting that neurological systems in the retina exert control over motor function as it relates to neurological conditions such as PD. While previous research has explored the effects anti-PD drugs [1,30,31], the present study extends this work further to include mechanisms of drug-induced PD and drug addiction [44]. This is important for two reasons. Firstly, both of these conditions are believed to involve the DA-positive reward system underlying the dopamine dysregulation syndrome of PD [41,45] and the mechanism of action in drug addiction [36,42]. That IVIT haloperidol slows motor response time suggests that the retina may be affected by systemic drugs, and through this be functionally tied to deep brain systems. In this regard, the importance of these results in relation to the action of antipsychotics and drugs for addiction opens new avenues for exploring the etiology, progression and treatment of these disorders [46,47]. Furthermore, that intervention at the level of the retina can alter behavioural control outside of the NSD illustrates how these findings challenge the traditional understanding of motor control. Exploration of pathways using electrophysiological and imaging techniques could provide deeper understanding of connections between visual and motor pathways underlying the etiology of PD and other neuropsychiatric disorders.

## Data Availability

The data presented in these studies are available upon reasonable request to the corresponding author and the decision to release data is governed by creative rights held by the author.
